# Delay in presentation to hospital for childhood cataract surgery in India

**DOI:** 10.1038/s41433-018-0176-2

**Published:** 2018-07-30

**Authors:** Sethu Sheeladevi, John G. Lawrenson, Alistair Fielder, Ramesh Kekunnaya, Rahul Ali, Rishi R. Borah, Catherine Suttle

**Affiliations:** 1 Division of Optometry and Visual Science, City, University of London, London, UK; 2 Child Sight Institute & Jasti V Ramanamma Children’s Eye Care Centre, L V Prasad Eye Institute, Hyderabad, India; 3 Orbis International, Haryana, India

## Abstract

**Purpose:**

Cataract is one of the major causes of avoidable visual disability in children and the aim of this study was to investigate the age at which children with cataract present for surgery at tertiary hospitals across India.

**Methods:**

A prospective multicenter study collected data from 9 eye hospitals in 8 states in India. All children admitted for cataract surgery between Nov 2015 and March 2016 were considered eligible. Parents were interviewed at the hospital by trained personnel and socio demographic information, age at diagnosis and at surgery and the relevant clinical data were obtained from the medical records. Mean age, age range at surgery were used and performed logistic regression analyses.

**Results:**

Parents of 751 consecutive cases were interviewed, of which 469(63%) were boys and 548 (73%) were from rural areas. Cataract was bilateral in 493 (66%) and unilateral in 258 (34%); of the unilateral cases, 179 (69%) were due to trauma. The mean age at surgery for ‘congenital’ and ‘developmental’ cataract was 48.2 ± 50.9 and 99.7 ± 46.42 months, respectively and the mean age was lower in the southern region compared to other regions. Children with 2 or more siblings at home were five times more likely to undergo surgery within 12 months (OR, 4.69; 95% CI: 2.04–10.79; *p* = < 0.001).

**Conclusions:**

Late surgery for childhood cataract remains a major challenge and the factors determining this issue in India are pertinent also to several other countries and need to be addressed for every child with cataract to achieve full visual potential.

## Introduction

Cataract is an avoidable cause of childhood vision impairment and blindness. Globally it is responsible for 5–20% of blindness in children [1] and is a priority for the VISION 2020: Right to Sight Initiative. A recent systematic review on the global burden of childhood cataract reported prevalence estimates ranging from 0.32 to 22.9 per 10000 children [2]. Although a treatable condition, previous research from India indicates that about half of the children in schools for the blind suffer from potentially preventable and/or treatable conditions with cataract being one of the leading causes [3].

Cataract in early childhood can be clinically classified as either *congenital* or *developmental* the former deemed to have greater impact on vision. This categorization is widely applied but is imprecise and unsatisfactory since it is almost impossible to know the age of onset of childhood cataract with most occurring during fetal life [4], while in many cases the cataract becomes apparent later in childhood with the precise age of onset remaining unknown. However, these terms are used in many countries to provide a clinical dichotomous indication of impact on vision, and are used as such here.

The developing visual system requires appropriate visual experience during infancy and early childhood. If compromised by cataract, bilateral or unilateral [5] form deprivation amblyopia results which affects the child’s educational [6] and psychosocial [7] development. Thus, while treatment of adult cataract is effective for visual rehabilitation at any age, a visually significant cataract that occurs in infancy and early childhood must be treated as early as possible, and requires long-term follow-up. In particular, unilateral visual deprivation in infancy results in more severe visual acuity deficits than bilateral deprivation, and this is related to the fact that in the unilateral condition visual development of the affected eye occurs in competition with an unaffected eye [8].

The time at which treatment of congenital cataract is most effective is within the first six to eight weeks after birth for unilateral cases [8], and within the first 14 weeks, for bilateral cases [9]. Previous reports suggest that late diagnosis limits the scope for successful outcomes [10, 11].

In developed countries, routine screening of all babies in the neonatal period facilitates early recognition of any lens opacity and timely surgical intervention [12]. Despite this in the UK even with routine examination only half of all cataracts in children are diagnosed within the first year of life [10, 11]. However, in developing countries, where neonatal eye screening is not routine as part of the health system, cataract surgery in children is delayed due to late recognition with correspondingly later presentation at hospital for treatment [13]. The possible reasons for delayed recognition and treatment vary geographically and include lack of new born screening programs, lack of knowledge among the parents /carers including the belief that nothing can be done [14] and limited accessibility to specialist centres.

Late presentation for surgery, inadequate follow-up and poor post-operative visual outcome in paediatric patients remain challenging in low and lower middle income countries [15, 16].

There have been few large-scale population-based studies investigating delay in paediatric cataract surgery [16–18]. Retrospective data from India indicate that only 50% of children with cataract present for surgery without delay [15]

India has the second largest population in the world and has diverse demographics including wide variations in the availability and utilization of health services as well as availability of health care resources between states [19]. Eye care is provided in different settings; including government hospitals, and non-governmental organizations (NGOs). The latter comprise of ‘not-for-profit’ and private hospitals, and each of these has different policies and practices regarding the fees structure. For example, government provision is free to all users, while not- for-profit hospitals charge fees on a sliding scale based on each individual’s ability to pay, and private hospitals charge relatively high fees, which applies to all patients [20]. Overall, 65% of the eye care services in India are currently offered through NGOs, including not-for-profit and private hospitals [21].

Currently, no prospective data are available on the age at presentation for childhood cataract surgery across India.

The main aim of this study was to investigate the age at recognition and presentation for cataract surgery among children at not-for-profit hospitals located across India and to identify the socio demographic factors associated with any delay in undergoing surgery.

## Methods

This study was approved by the School of Health Sciences Research Ethics Committee at City, University of London and by the Institutional Review Boards of all 9 participating hospitals in India and the principles of the Declaration of Helsinki were followed throughout. The data were collected at the locations shown below (4 to 9 are Orbis International supported partner centers) using a prospective observational study design. All the 9 hospitals are not-for-profit NGOs, and provide free services to the individuals who are unable to pay for the services.

1. L V Prasad Eye Institute (LVPEI), Hyderabad, Telangana
2. L V Prasad Eye Institute (LVPEI), Visakhapatnam, Andhra Pradesh
3. L V Prasad Eye Institute (LVPEI), Vijayawada, Andhra Pradesh
4. Dr. Shroff’s Charity Eye Hospital, New Delhi
5. PBMA’s H V Desai Eye Hospital, Pune, Maharashtra
6. Sadguru Netra Chikitsalaya, Chitrakoot, Madhya Pradesh
7. Little Flower Hospital & Research Centre, Angamaly, Kerala
8. Vivekanand Mission Ashram, Netra Niramaya Niketan, Haldia, West Bengal
9. Sri Sankaradeva Nethralaya, Guwahati, Assam

A sample size of 720 was calculated based on an estimated late presentation rate of 50% [15], with 5% precision, 95% CI and a refusal rate of 20%. We prospectively approached the parents or carers of all children (aged < 18 years) admitted for cataract surgery between 16th Nov to 5th March 2016, and those who gave consent were enrolled.

At each center, a member of staff was trained for this project and they conducted the interview with the parents in the local language. A structured questionnaire was developed based on literature review with the aim of estimating the age of recognition and presentation for childhood cataract surgery. It was piloted before the main study and after minor modifications the finalized questionnaire was translated to Telugu, Hindi, Malayalam, Marathi, Bengali and Assamese languages and back-translated to ensure accuracy and consistency of content.

The study team at each hospital identified children admitted for surgery and approached the parents or care providers individually with the written information about the study for obtaining consent. As a quality check, all of the enrollments were cross-checked with the medical records by the principal investigator (SS) and 10% of the interviews were observed in person by SS in each of the nine hospitals.

### Case definitions

Paediatric ophthalmologists at the study sites followed uniform criteria to diagnose cataract in children. Cataract was classified as congenital if recognized at or within 2 months of birth and / or if accompanied by nystagmus with no other pathology, or classified as developmental if the cataract was recognized after two months from birth, was zonular in nature and without nuclear involvement. In the case of total cataract, the decision was primarily based on the history of the onset of the cataract and if associated with trauma it was classified as traumatic cataract. Cases of traumatic cataract were excluded from the analysis.

### Late presentation

For all congenital cataracts, presentation for surgery more than 12 months from birth was defined as late presentation. Although it is recommended to operate earlier than this for better visual outcomes [9], this definition was used for pragmatic reasons. Definition of late presentation for surgery was more complex in developmental cases. The cataract many have existed for some time prior to recognition, so the period of delay has greater uncertainty in developmental than congenital cases. For this reason no attempt was made to calculate delay in presentation in developmental cataract and only the mean ages at recognition and at surgery were reported in these cases.

### Statistical analysis

These data were analyzed using SPSS version 22. *Χ*^2^ test was used to identify associations between the gender and a *p* value less than 0.05 was considered statistically significant. One way ANOVA test was used to determine the differences between the regions and univariate and multiple logistic regression analyses were used to look for associations between time to presentation for surgery for congenital cataract and a range of demographic factors. Results were presented as odds ratios (ORs) with 95% confidence intervals (CIs).

## Results

Nine hospitals representing 5 geographic regions (South (*n* = 4); West (*n* = 1); Central (*n* = 1); East (*n* = 2) and North (*n* = 1)) in eight states across India participated in this study. A total of 780 children were admitted for cataract surgery during the study period, of which 751 (96%) consented to participate in the study. Of the 751 subjects, 469 (62.5%) were boys and 282 (37.5%) were girls. The mean age of the participants at hospital admission was 83.6 months (SD 56.0) including traumatic cataract.

According to the study definition, 289 (38%) of the children presented with congenital cataract, 283 (38%) children with developmental cataract, including 14 (2%) children were diagnosed with cataract caused by infections or diabetes and 179 (24%) children with a history of trauma. Bilateral cataract was present in 493 (86%) children, and unilateral cataract was present in 79 (14%) children.

As shown in Table 1, most of the socio demographic factors were not found to be associated with the genders.

**Table 1:** Socio demographic details of the study participants by gender

Variables	Boys *n* (%)	Girls *n* (%)	*P* value
Cataract type			
Congenital	167 (49.7)	122 (51.7)	0.67
Developmental	169 (50.3)	114 (48.3)	
Laterality			
Bilateral	291 (86.6)	202 (85.6)	0.81
Unilateral	45 (13.4)	34 (14.4)	
Family History of Childhood cataract	34 (10.1)	36 (15.3)	0.07
Treatment category			
Paying	175 (52.1)	118 (50.0)	0.67
Non paying	161 (47.9)	118 (50.0)	
Place of birth			
Hospital	234 (69.6)	154 (65.3)	0.28
Home	102 (30.4)	82 (34.7)	
Residential location			
Urban	54 (16.1)	34 (14.4)	
Semi-rural	47 (14.0)	29 (12.3)	0.68
Rural	235 (69.9)	173 (73.3)	
Region			
South India	139 (41.4)	111 (47.0)	
Central India	102 (30.4)	72 (30.5)	
West India	18 (5.4)	12 (5.1)	0.54
North India	36 (10.7)	19 (8.1)	
East India	41 (12.2)	22 (9.3)	
Parental consanguinity	88 (26.2)	66 (28.0)	0.70
Father’s education			
No education	87 (25.9)	83 (35.2)	
School education	202 (60.1)	118 (50.0)	0.04
University education	47 (14.0)	35 (14.8)	
Mother’s education			
No education	113 (33.6)	91 (38.6)	
School education	188 (56.0)	110 (46.6)	0.06
University education	35 (10.4)	35 (14.8)	
Father’s occupation			
Daily Laborer	235 (69.9)	170 (72.0)	
Formal employment (government / private sector)	67 (19.9)	36 (15.3)	0.27
Others	34 (10.1)	30 (12.7)	
Mother’s occupation			
Not working	256 (76.2)	174 (73.7)	
Daily Laborer	69 (20.5)	48 (20.3)	0.31
Formal employment (government / private sector)	11 (3.3)	14 (5.9)	
Family’s reported monthly income			
(£1 = INR 80)			
<INR 5000	146 (43.5)	110 (46.6)	0.12
INR 5001- INR 20,000	171 (50.9)	104 (44.1)	
>INR 20,001	19 (5.7)	22 (9.3)	

*INR* Indian rupees

**Fig. 1:**
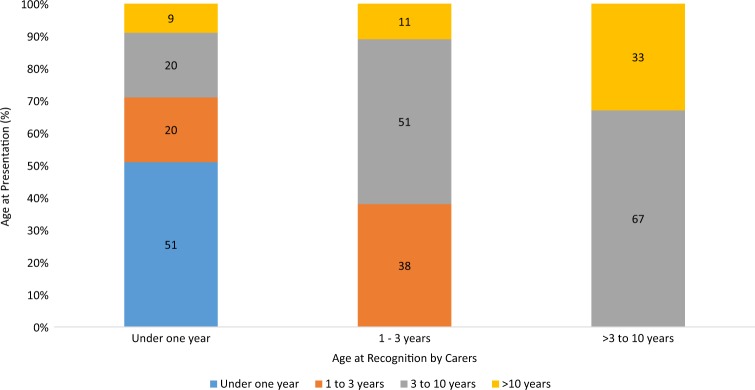
Comparison of age at recognition and the age at presentation for surgery for childhood cataract (Proportion of children). *X* axis indicates the age at recognition by the carers. Each column represents the proportion of children recognized during the time period and different colors indicate the time at which surgery was completed

In 227 (40%) of the children with either congenital or developmental cataract, the condition was recognized before 1 year of age and of these 71 (12.4%) were recognized during the neonatal period (0–28 days). About half (51%) of the 227 underwent surgery within 1 year of birth, 19.4% between 1 and 3 years, 20.3% between 3 and 10 years and around 9% after 10 years of age (Fig. 1).

Mean age for childhood cataract surgery (excluding the traumatic cataract) was 74.6 months (SD 55.8); congenital 48.2 (SD 50.9); developmental 99.7 (SD 46.42). The comparative data on age at recognition and their respective mean age at surgery, laterality for both congenital and developmental cataract is shown in Table 2.

**Table 2:** Age at recognition and the mean age at surgery for congenital and developmental cataract

Age (in months) at which eye problem was recognized	Mean and age range for childhood cataract surgery (in months)					
	Congenital cataract			Developmental cataract		
	*n* (%)	Unilateral	Bilateral	*n* (%)	Unilateral	Bilateral
≤12	111	7.3	6.2	5		7.5
	(38.4)	(2.2–12.0)	(1.2–12.0)	(1.9)	0	(3.6–12.0)
>12–36	58	23.5	22.4	18	32.7	29.7
	(20.1)	(18.0–36.0)	(13.1–36.0)	(6.7)	(30.6–33.9)	(16.8–36.0)
>36–120	79	79.5	74.2	140	69.7	74.8
	(27.3)	(37.1–108.0)	(36.3–109.3)	(52.0)	(48.0–114.9)	(36.7–114.4)
>120	41	144.4	147.3	106	145.4	150.5
	(14.2)	(120.0–178.7)	(120.0–198.7)	(39.4)	(120–180.0)	(120–194.5)
All cases	289	51.8	47.7	269	113.2	97.2
	(100)	(2.2–178.7)	(1.2–199.0)	(100)	(30.6–180.0)	(3.6–194.5)

In half of the subjects (50%), a parent recognized the eye problem and in 81 (14.2%) it was recognized by another relative including grandparents or older siblings. In 149 (26.0%) cases, it was recognized by health workers and in 56 (10%) of cases the cataract was recognized by school teachers.

**Fig. 2:**
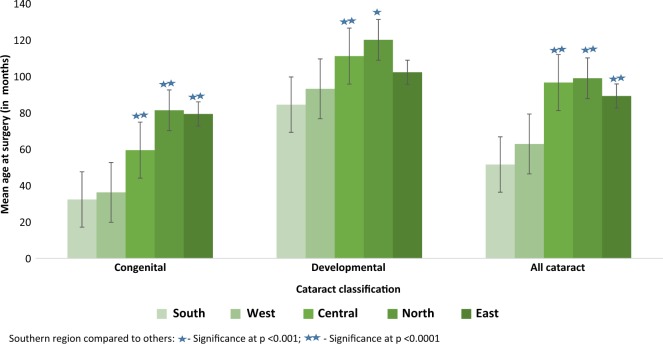
Mean age (±standard error) of childhood cataract surgery across India.

The mean age at surgery for congenital cataract in the Southern, Western, Central, Eastern and Northern regions was 32.4, 36.3, 59.5, 79.4 and 81.4 months, respectively, while the corresponding figures for developmental cataract were 84.5, 93.2, 111.2, 102.3 and 120.1 months. The mean age at presentation was low in the Southern and Western regions compared to others (Fig. 2).

In the univariate regression model for congenital cataracts, the age at presentation for late surgery (more than 12 months) had statistical correlation with location (e.g. rural), geographical region, parental education and age, number of siblings at home and father’s occupation (all *p* = < 0.01). In the multivariate model, geographical region and the number of siblings at home were associated significantly with late surgery. Children with two and more siblings at home were almost five times more likely to undergo surgery within 12 months of age than those with one or no siblings (OR: 4.69; 95% CI: 2.04–10.79; *p* = < 0.001) (Table 3).

**Table 3:** Multivariate Logistic regression analysis of underlying factors associated with surgery later than 12 months for congenital cataract

Factors	Univariate		Multivariate	
	OR (95% CI)	*p* value	OR (95% CI)	*p* value
Gender (Boys vs Girls)	0.83 (0.51–1.34)	0.44	1.38 (0.77–2.45)	0.28
Birth place (Hospital vs. home)	0.20 (0.09–0.43)	< 0.001	2.23 (0.92–5.45)	0.08
Location (Rural vs urban)	1.31 (0.69–2.44)	0.41	1.31 (0.60–2.82)	0.49
Region (South & West vs. North & East)	3.27 (1.93–5.55)	< 0.001	1.53 (0.78–3.02)	0.22
Treatment category (Paying vs. nonpaying)	2.37 (1.46–3.85)	< 0.001	1.53 (0.78–3.05)	0.22
Laterality (Bilateral vs. unilateral)	1.11 (0.53–2.35)	0.79	1.06 (0.43–2.56)	0.91
Family history with childhood cataract	1.97 (0.92–4.21)	0.08	1.21 (0.46–3.15)	0.69
Parental consanguinity	1.16 (0.69–1.94)	0.56	1.08 (0.56–2.11)	0.81
Father’s age (<30 years vs. ≥30 years)	2.92 (1.79–4.78)	< 0.001	1.88 (0.95–3.71)	0.07
Mother’s age (<30 years vs. ≥30 years)	3.25 (1.76–6.00)	< 0.001	1.49 (0.67–3.29)	0.33
Father’s education (No education vs. any education)	3.29 (1.85–5.86)	< 0.001	1.55 (0.68–3.51)	0.29
Mother’s education (No education vs. any education)	2.71 (1.55–4.76)	0.001	1.40 (0.63–3.11)	0.41
Father’s occupation (Laborers vs. all others)	2.25 (1.34–3.79)	0.002	1.30 (0.61–2.79)	0.49
Mother’s occupation (Not working vs. working)	1.15 (0.69–1.92)	0.60	1.11 (0.49–2.47)	0.78
No of siblings (Only child vs. 2 or more)	8.55 (4.15–17.6)	< 0.001	4.26 (1.84–9.85)	< 0.001
Family’s reported income (<INR 5000 vs. >INR 5001; £1 = INR 80)	2.86 (1.68–4.85)	< 0.001	1.85 (0.89–3.83)	0.09

## Discussion

The mean age at surgery for congenital cataract was 4 years and for developmental cataract it was 8 years. More than two-thirds of the congenital cataracts were recognized within 1 year of birth, around two-thirds of all cataracts were recognized by family members and in half of the cases it was by a parent. The reason for increased mean age at surgery in developmental cataract, may be due to delay in recognition or may be related to the level of visual loss, with some cataract being more visually significant than others.

Overall, half of all cataract surgeries in the present study occurred later than 12 months from birth, and this is consistent with findings of a retrospective study from India [15]. However, the proportion of children undergoing surgery within 6 months from birth was lower (16%) in the current study than has been reported in China (28%) [13]. Similarly, another study from China indicates that a mean age at surgery for congenital cataract of 27.6 [22] vs. 48.2 months in the present study, and for all childhood cataract a retrospective study from Southern India found mean age at surgery of 53.0 [23] vs. 74.6 months in the present study. The higher mean age in the present study may reflect the fact that the data were drawn from different regions in the country, while the data showing earlier surgery were from the South. For example, we found the mean age at surgery to be higher in the Central and the combined Northern and Eastern regions of the country than in the Southern region, by factors of 2 and 2.5 respectively. These disparities are known [24] and the Southern region, in particular, has a higher number of not-for-profit eye hospitals and the active implementation of community eye care activities across the region may have led to greater awarenesss of the need for early surgery.

Even though more than two-thirds of cases in this study were recognized within one year, these findings indicate that there are impediments to early surgery for childhood cataract in India.

Early treatment of childhood bilateral and unilateral cataract is important to optimize vision in life and to minimize amblyopia. Visual disability in untreated bilateral cataract has an impact on general development, including educational [25] and social aspects. As outlined earlier (see “Introduction” section), visual development is more adversely affected by unilateral than bilateral cataract in infancy. Specifically, visual acuity deficits are more severe in congenital unilateral than bilateral cataract, since the visual deprivation caused by the cataract occurs during a critical period for visual development and in the unilateral case, the resulting abnormal development (amblyopia) is exacerbated by competition from the unaffected eye. It has been shown that early surgery improves the visual outcomes in both unilateral and bilateral cataracts [26]. It has been previously reported that congenital cataract operated after 1 year was associated with poorer visual outcomes [23] and in the current study 40% of all congenital cataract cases underwent surgery >3 years from birth. However, it is worth noting the strong potential for unilateral cataract to have severe negative effects on visual development, and the need for early treatment (while the visual system has plasticity) in these cases.

Prevalence of bilateral and unilateral childhood cataract has previously been reported to be similar [2, 9]. However, in the current study, 86% of the children presented with bilateral cataract and only 14% presented with unilateral cataract. This is an important finding since it suggests a low rate of presentation of unilateral cataract, and a high proportion of cases unpresented and untreated, at risk of impaired visual development and permanent visual deficit in one eye.

Globally, no gender difference is reported in the prevalence of childhood cataract [2]. However, in the current study, in all regions of India, more boys presented for surgery than girls (60 vs. 40%), which is similar to previous reports in Africa [27] and in India [28]. This is supported by published evidence from India that boys are much more likely than girls to undergo hospital based treatment, particularly in economically poor families [29].

The differences highlighted here, including gender imbalance at baseline, laterality and regional delay in accessing childhood cataract services need to be bridged to achieve equitable access to health care, which is considered crucial for reaching many of the Sustainable Development Goals. (http://www.un.org/sustainabledevelopment/)

There are challenges in defining childhood cataract. One widely used definition is the age at onset: a congenital or infantile cataract is said to present within the first year of life [12], whereas cataract presenting between 1 and 10 years of life is classified as developmental or juvenile cataract. For example, Mwende [18] and You et al. [13] defined congenital cataracts as those recognized by carers or which presented at the hospital when the child was below the age of 1 year. All those recognized after 12 months and not due to trauma were defined as developmental cataract. However, as outlined earlier, childhood cataract that appears to not be congenital (not apparent at or within a certain time window after birth) is generally categorized as developmental, but the distinction between these two types is unclear. Clarity in defining and determining the time to surgery in childhood cataracts is essential to address any access issues in these cases.

In the present study, if we assume all congenital cataracts categorized by the clinicians are truly congenital (from birth), only one-third of the unilateral congenital cataract cases completed surgery within 1 year. And only one-fifth of all congenital cataracts were surgically treated within 1 year with the remaining 80% treated later, from 1 to 10 years. In about half of the cases, the cataract was recognized by a parent or carer, so in these cases lack of parental awareness did not cause any delay, instead, other factors such as accessibility to the surgical centres may be responsible. It was significant to note the association of two or more siblings in the home with early presentation for surgery. Perhaps, these parents were able to compare the developmental milestones and the behavior of the child with cataract with other siblings and identify the problem earlier and access treatment.

To our knowledge, this is the first multi-center study aimed at estimating the actual age at presentation for childhood cataract across India. Although the data were obtained from several geographical regions, all of the hospitals included in the study were not-for-profit, and therefore the findings cannot be generalized to the entire population. For example, parents using the private hospitals for their child’s treatment may be a different socio-demographic group. Nonetheless, our study has highlighted that delayed presentation for surgery remains a significant problem in India for children with congenital or developmental cataract. Understanding the reasons for delayed presentation and /or surgery will provide valuable insights to reduce the time gap between the onset of vision impairing cataract and surgery. Reducing these gaps will improve the visual outcomes of childhood cataract surgery and thus contribute to achieving one of the main priorities of VISION 2020: The Right to Sight Initiative, reducing blindness and vision impairment in children.

## Conclusion

This study confirms that time-to-surgery for childhood cataract remains a major problem in India. The factors contributing to this include gender, laterality and also local health service factors. Though these factors are identified in India, they are likely to be pertinent to several other countries. Region-specific efforts are required from all stakeholders in the community to ensure children with cataract receive timely surgical services to reduce blindness and vision impairment caused by cataract so that they may achieve their full visual potential. Further work is underway to examine the barriers to treatment for childhood cataract in India and how these might be removed.

### Summary

#### What was known before

- Presentation for childhood cataract is often delayed in developing countries. Age at surgery is important factor for better post-operative outcome.

#### What this study adds

- Time gap between age at recognition and age at surgery for congenital cataract.
- Mean age at recognition and at surgery for congenital and developmental cataract in children. socio demographic factors associated with this delayed presentation.
